# Clinical forms of 5 cases of circumcised penile cancer in immunocompetent subjects in Abidjan (Ivory Coast)

**DOI:** 10.1186/s12610-025-00253-6

**Published:** 2025-02-06

**Authors:** Evrard Kouame Yao, Abraham Hognou Yao, Donafologo Daouda Yeo, Tawakaltu Adebayo, Michel Tuo, Freddy Junior Zouan, Noel Coulibaly

**Affiliations:** 1 Urology Department, Treichville University Hospital, Abidjan, Ivory Coast; 2 Department of Surgery and Specialties, University Felix Houphouet Boigny, Abidjan, Ivory Coast; 3 Surgery Department, Abidjan Military Hospital, Abidjan, Ivory Coast

**Keywords:** Biopsy, Budding mass, Ulceration, Biopsie, Masse bourgeonnante, Ulcération

## Abstract

**Context:**

Malignant tumours of the penis are rare. Their incidence varies around the world and increases with age. Treatment is essentially surgical. Surgery may be conservative or radical.

We report the anatomoclinical aspects of 5 cases of penile cancer observed in our department.

**Case presentations:**

A 44-year-old patient with a circumcised penis, diagnosed with a fusosarcoma (a rare type of sarcoma) of the penis. Three patients (ages 61, 59, and 70) were diagnosed with squamous cell carcinoma, the most common type of penile cancer. These patients also had circumcised penises. The last case was a metastatic squamous cell carcinoma of the penis in a 68-year-old patient. All patients were immunocompetent and had a circumcised penis. They refused surgery in any form.

**Conclusion:**

The clinical aspects of penile cancer are polymorphous and misleading. Any bulging or indurated lesions on the penis should be biopsied.

## Résumé

**Contexte:**

Les tumeurs malignes du pénis sont rares. Leur incidence varie dans le monde et augmente avec l’âge. Le traitement est essentiellement chirurgical. La chirurgie peut être conservatrice ou radicale. Nous rapportons les aspects anatomocliniques de 5 cas de cancer du pénis observés dans notre service.

**Présentations des cas:**

Un patient de 44 ans avec un pénis circoncis, diagnostiqué avec un fusosarcome (un type rare de sarcome) du pénis. Trois patients (âgés de 61, 59 et 70 ans) ont reçu un diagnostic de carcinome épidermoïde, le type le plus courant de cancer du pénis. Ces patients avaient également des pénis circoncis. Le dernier cas était un carcinome épidermoïde métastatique du pénis chez un patient de 68 ans.

Tous les patients étaient immunocompétents et avaient un pénis circoncis. Ils ont refusé la chirurgie sous quelque forme que ce soit.

**Conclusions:**

Les aspects cliniques du cancer du pénis sont polymorphes et trompeurs. Toute lésion bombée ou indurée, sur le pénis, doit être biopsiée.

## Background

Penile cancer is rare in regions like the European Union and the United States, affecting approximately 1 in 100,000 men. It is more common in other parts of the world, accounting for 1–2% of all cancers in some regions [1, 2]. The incidence of penile cancer increases with age, suggesting a possible age-related risk factor. The human papillomavirus (HPV) infection plays a significant role in the development of penile cancer HPV infection is found in 70 to 100% of precancerous penile lesions, known as penile intra-epithelial neoplasia (PIN), which is a precursor to cancer. HPV is also present in 30 to 40% of penile cancer cases, indicating its role in the carcinogenesis process [2, 3].

Malignant tumours of the penis are a rare tumour in Ivory Coast. Akassimadou et al. in Bouake (Ivory Coast) described 3 cases in 5 years [4].

There are many treatment options. Surgery is the gold standard. Treatment methods range from conservative surgery for small tumours to radical surgery for large tumours [5].

There are many different clinical forms. They are often confused with benign skin lesions.

In our context, patients are diagnosed in the locally advanced stages. However, radical surgery is difficult for patients to accept. This leads to loss of follow-up.

We report 5 cases of penile cancer with a focus on clinical forms.

## Case reports

### Case 1

**Fig. 1:**
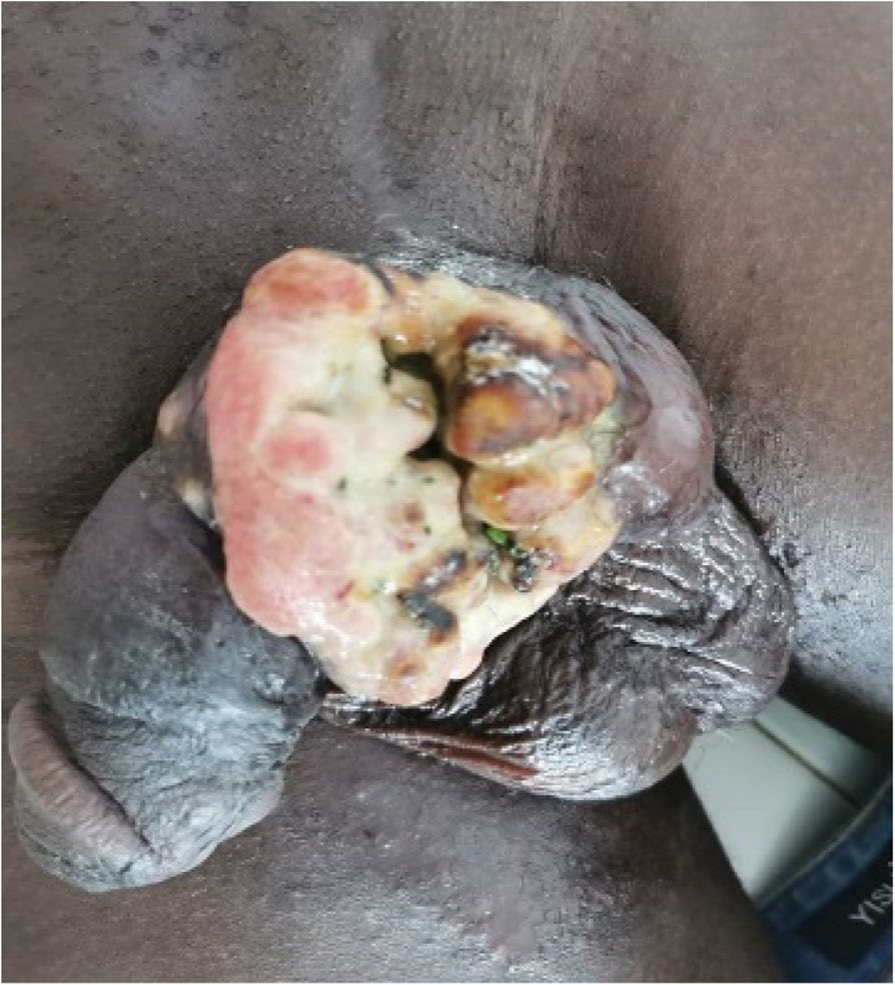
Ulcerating mass at the base of the penis with induration at its base

Mr G.H, a 44-year-old patient. He had no major pathological history. He presented with an ulcerating mass on the penis. This painless mass had been developing for 3 months. There was no fever. On physical examination, an ulcerating, budding mass 7 cm long was noted at the base of the penis (Fig. 1). The mass bled on contact. There was also induration of the homolateral corpus cavernosum. There were no superficial inguinal adenopathies.

Biopsy revealed a fuso baso cellular sarcoma of the penis.

After an extension assessment (MRI, CT scan), amputation of the penis with lymph node dissection was recommended. The patient refused surgery. We saw him again 3 weeks later. He presented with haemorrhagic shock following massive bleeding from the ulcerating lesion. He benefited from resuscitation measures.

The patient died after one month due to progression of the cancer.

### Case 2

**Fig. 2:**
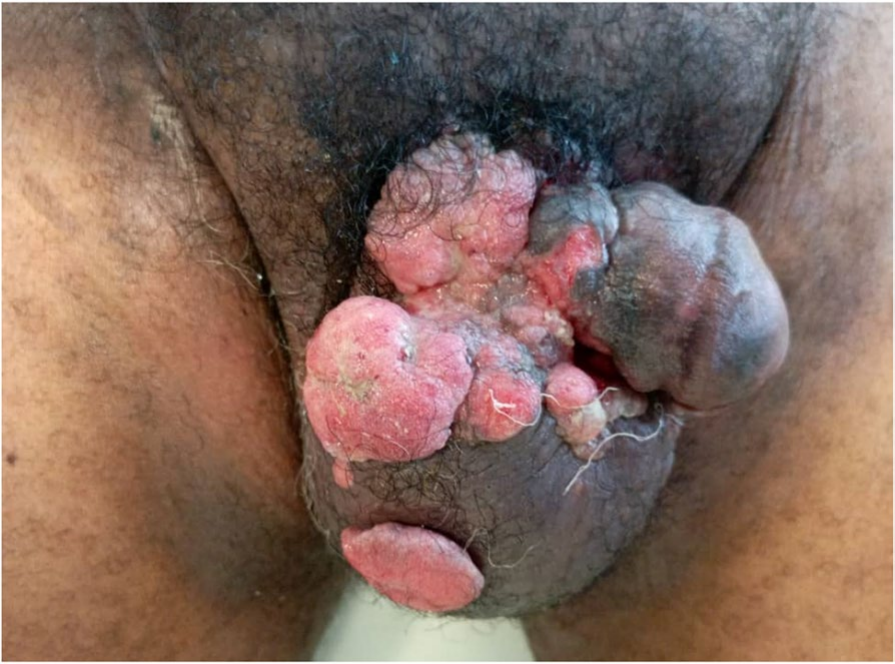
Multifocal ulcerating mass of the scrotum and root of the penis

Mr T.K was a 61 year old patient with no previous history of major disease. He came to us with ulcerating lesions on the penis and bursa that had been progressing for 3 months. On physical examination, the patient was circumcised, and the lesions were all over the penis, it was princesse, on the right lateral surface and the scrotum (Fig. 2). There was no induration of the corpora cavernosa or superficial inguinal adenopathy. Biopsy revealed squamous cell carcinoma of the penis. Emasculation was indicated. The extension (pelvic MRI, thoraco-abdominal CT scan) led to the conclusion of T4N0M0 (scrotal involvement). The patient refused surgery and all systemic treatments. After the patient was discharged at his request, he was lost to follow-up.

### Case 3

**Fig. 3:**
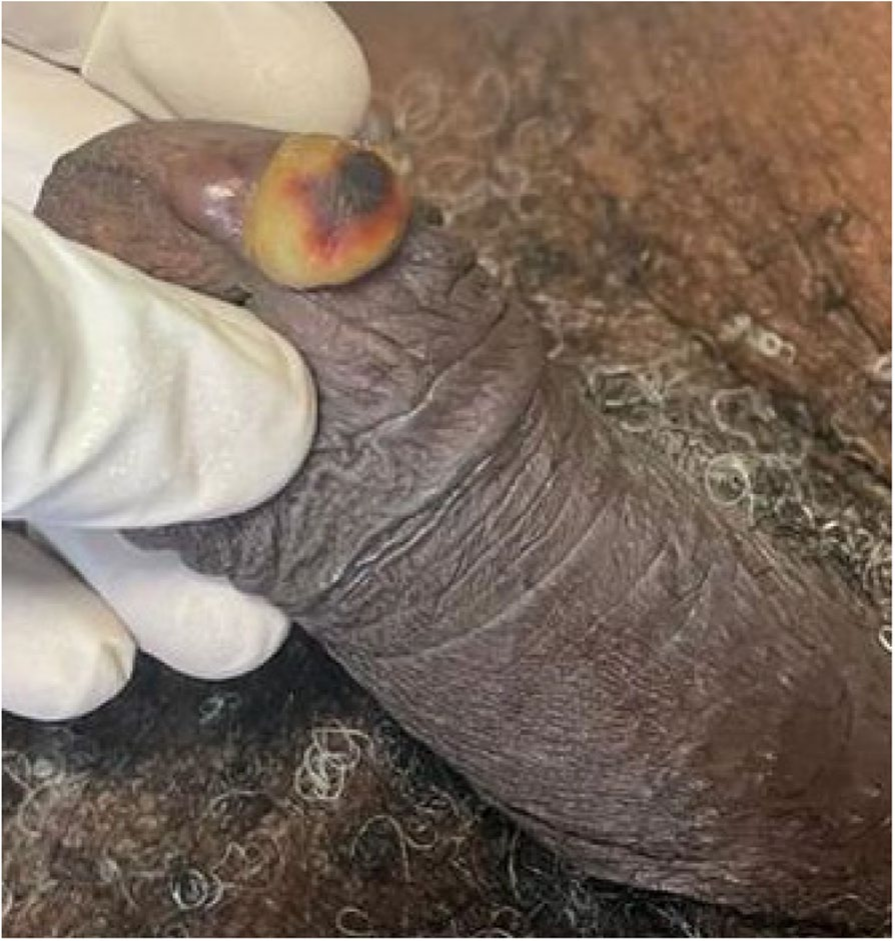
Small mass on the ventral surface of the penis which may be confused with a benign lesion

Mr A.Y, aged 59. He is hypertensive,diabetic and complies with treatment. He came to us with a mass on the ventral surface of the glans penis. This had been developing for 6 months. Physical examination revealed a circumcised patient and a hard, rounded mass on the ventral surface of the glans. The mass was painless and associated with induration of the right corpora cavernosa and urethra (Fig. 3). No superficial adenopathy was noted. Biopsy revealed a squamous cell carcinoma of the penis. The extension was T2N0M0. Partial penectomy was recommended. The patient refused and opted for plant extracts. He was readmitted after 3 months in a state of septic shock. He died in the intensive care unit.

### Case 4

**Fig. 4:**
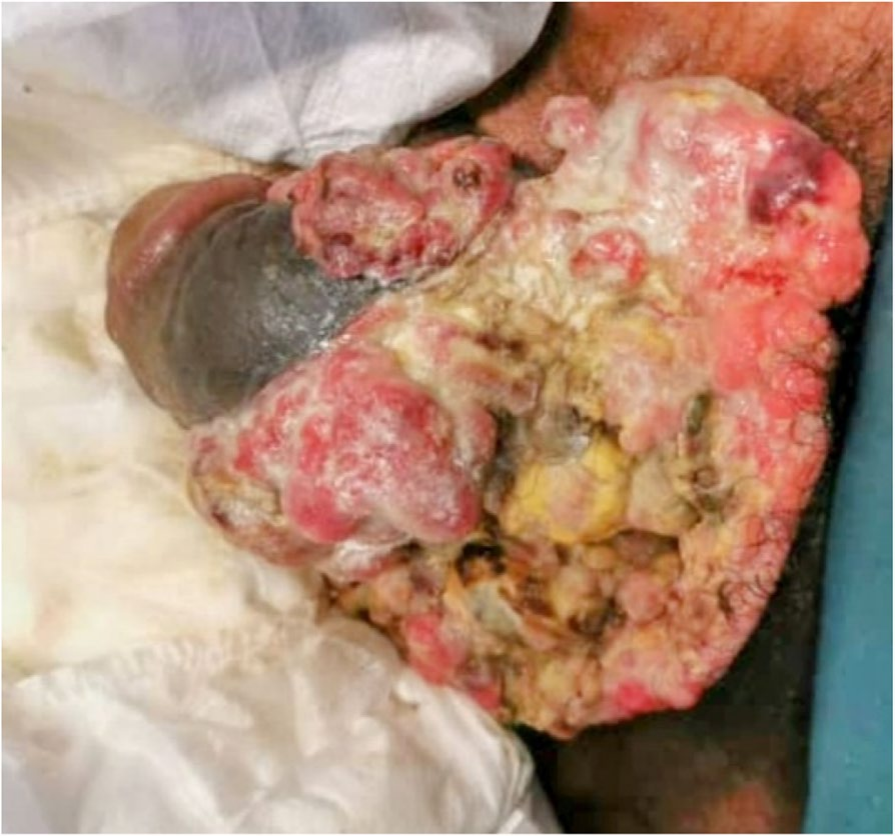
Large bulging mass of the penis with contralateral deviation of the penile axis

Mr. O.A., 68 years old. was being treated for a prostate adenoma and had been on alpha blockers for approximately three years. He consulted for an ulcerating and necrotizing lesion on his penis, which had been developing over the past year. Despite seeking treatment with plant extracts for several months, the lesion continued to progress. He had previously received unspecified treatment from a urology department but refused surgery when it was indicated. On physical examination, the patient appeared emaciated and experienced intense bone pain. Additionally, he presented with 4/5 paraplegia due to spinal cord compression. The patient had a circumcised penis, and upon examination, an extensive wound was observed on the ventral surface of his penis and scrotum (Fig. 4).

A biopsy of the lesion confirmed the diagnosis of squamous cell carcinoma (SCC) of the penis. The extension concluded that the cancer was staged as T4N1M1. Mr. O.A. received systemic treatment (chemotherapy). However, due to his advanced stage and refusal of surgery, the prognosis was poor. The patient passed away approximately one week after admission. This case illustrates a late-stage diagnosis of penile squamous cell carcinoma with metastasis and severe complications. The patient had multiple comorbidities, including prostate adenoma and spinal cord compression, and despite initial treatment attempts with plant extracts, the cancer progressed rapidly. The patient’s refusal of surgery and advanced cancer likely contributed to the rapid deterioration and poor prognosis.

### Case 5

**Fig. 5:**
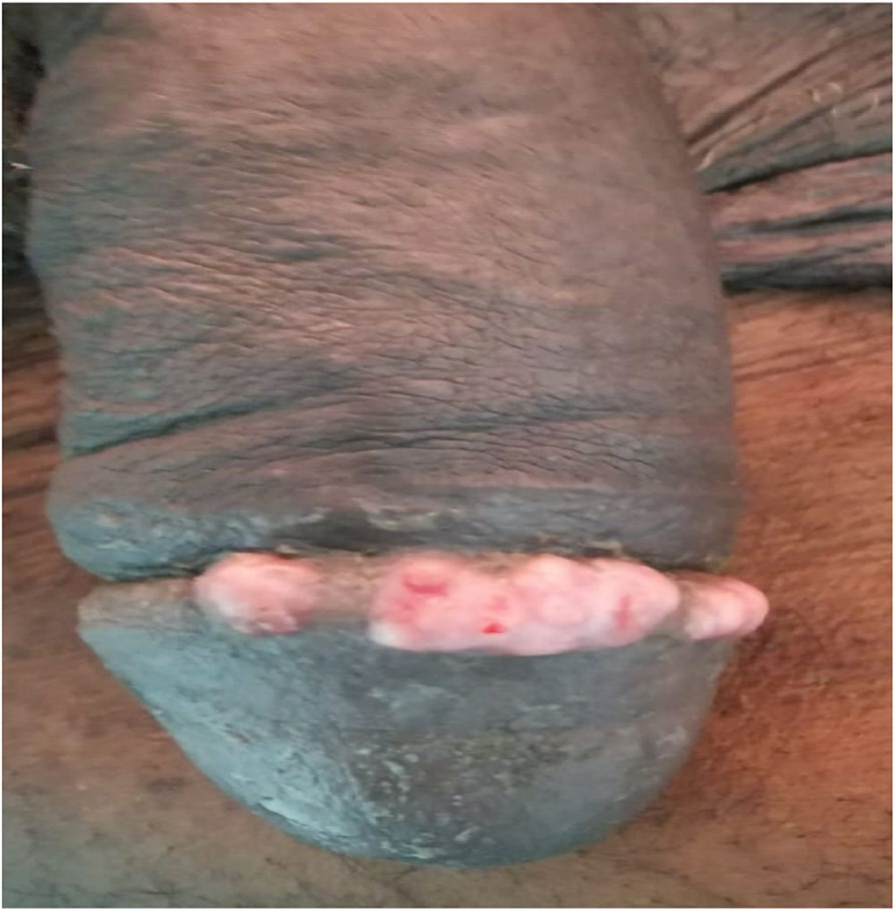
“Rosary” lesions of the crown of the penis that can be confused with macerated lesions

Mr K.A was 70 years old. He had a history of prostatic adenomectomy. He has being treated for metastatic adenocarcinoma of the prostate. He presented with a papular lesion on the dorsal surface of the glans penis. This lesion was located on the crown of the glans. It was painless and non-haemorrhagic. There was bilateral induration of the corpora cavernosa down to the base of the penis (Fig. 5). There were painless mobile right inguinal adenopathies.

Biopsy confirmed the diagnosis of squamous cell carcinoma of the penis. It was classified as T3N1M0 after extension. After a positive lymph node cyto-puncture, chemotherapy was proposed and is currently under way. Local treatment will depend on the response to chemotherapy.

## Discussion

Penile cancer is rare, with studies from Côte d’Ivoire and Senegal confirming its low prevalence. In Senegal, penile cancer accounts for just 0.35% of all cancers [6, 7]. The patients in the report were relatively older, aligning with findings from Gueye et al. in Senegal, who found an average age over 50 years for penile cancer patients [6]. Early circumcision is suggested as a major protective factor. All the patients in this study were circumcised, which is consistent with the theory that circumcision reduces the risk of penile cancer [8, 9]. All our patients were circumcised. All patients tested negative for HIV, which is notable because HIV infection is a known risk factor for several cancers, including penile cancer, due to the associated immune suppression and increased susceptibility to infections like HPV [10, 11]. A significant clinical feature in this context was the delay in diagnosis, with all patients being diagnosed at advanced stages (locally advanced or metastatic). For three patients, the tumor had been developing for several months before seeking medical attention.

Typical Clinical Features: The clinical presentation is often a ulcerating, budding mass, with the base of the penis being a common site of involvement.

Amputation of the penis, though often a necessary procedure in advanced cases of penile cancer, is considered a mutilating treatment. In many African cultures, the penis is viewed as a symbol of masculinity and virility, which makes this option particularly difficult for patients to accept.

As demonstrated in the earlier case reports, patients frequently refuse surgery, including amputation, because of cultural and social perceptions of the penis as a central part of their identity. Squamous Cell Carcinoma (SCC) is the most common histological form of penile cancer, as is the case with most of the patients in this report [12].

For the basal Cell Carcinoma, while rarer, it is mentioned as another possible histological form that should be considered.

People need to be made aware of the advantages of early diagnosis in order to improve treatment.

All skin lesions on the penis and any induration of the corpus cavernosum should be investigated for penile cancer.

According to AFU recommendations, the treatment of penile cancer is essentially surgical, combined to a greater or lesser extent with chemotherapy in the event of lymph node involvement. Lymph node involvement can be clarified using certain imaging techniques such as PET scan [13].

However, our limited technical resources (no PET scan) do not allow us to apply these recommendations. We have therefore adopted local recommendations based on our daily practice. We consider a supra-centimetre node on MRI to be a potentially positive node. This supra-centimetre node allows us to indicate a cytopuncture.

The small number of cases, which does not allow an analytical approach to this series, is the main limitation of our study.

## Conclusion

The clinical aspects in this series are different from one another. some lesions have the clinical appearance of benign lesions. The ulcero-burgundy appearance with squamous cell carcinoma as the histological lesion is found in the majority of cases. We suggest a biopsy for any painless penoscrotal mass with a chronic course.

## Data Availability

No datasets were generated or analysed during the current study.

## Abbreviations

HIV: Human immunodeficiency virus
HPV: Human papilloma virus
MRI: Image by magnetic reasoning
